# Review on Major Challenges of Veterinary Service Delivery in Ethiopia

**DOI:** 10.1002/vms3.71208

**Published:** 2026-09-22

**Authors:** Abdu Muhammed, Mitiku Wamile

**Affiliations:** ^1^ Department of Animal Science Wollega University Shambu Ethiopia

**Keywords:** accessibility, diagnostic technologies, infrastructure, veterinary professional

## Abstract

**Background:**

Ethiopian veterinary services encounter several difficulties, including inadequate infrastructure, difficult accessibility, and financial restrictions. Inadequate veterinary clinics, diagnostic laboratories, and cold chain facilities, particularly in rural and pastoralist settings, negatively affect livestock health and production.

**Objectives:**

The objective of this review is to identify and analyze major challenges hindering effective veterinary service delivery in Ethiopia.

**Methods:**

This review was conducted by systematically searching electronic databases including PubMed, Google Scholar, Scopus, and the Web of Science for articles published between 2000 and 2025. Search keywords included ‘Ethiopia’, ‘veterinary service delivery’, ‘animal health’, ‘infrastructure’, ‘diagnostic laboratories’, ‘veterinary workforce’, ‘financial constraints’, and ‘disease surveillance’. Additional sources were identified from the reference lists of relevant articles and from grey literature.

**Results:**

The nation's access to and use of veterinary services are restricted by a severe shortage of qualified veterinary professionals (veterinarian‐to‐livestock ratio far below WOAH recommendations) as well as budgetary limitations. The underutilization of cutting‐edge diagnostic technologies, such as genomic and molecular tools, impairs the ability to monitor and respond to illnesses, especially zoonotic ones. This is compounded by a tiered laboratory system where only the national reference lab has routine molecular diagnostic capacity, and inadequate quality control and standardization in laboratories lead to unreliable results. Inadequate data exchange and management hamper coordination of responses to epidemics. The economy and animal health services in Ethiopia are impacted by a scarcity of veterinary specialists, exacerbated by brain drain. Low pay and lack of incentives discourage veterinarians from practicing in underprivileged regions. The high cost of veterinary treatment is a challenge for livestock owners, particularly in rural locations. Additionally, underutilization and poor animal health outcomes are caused by a lack of public awareness about veterinary services in rural areas.

**Conclusions:**

Veterinary service delivery in Ethiopia is constrained by infrastructure gaps, workforce shortages, financial limitations, weak diagnostic capacity, poor data management, and low community awareness. Addressing these challenges requires increased investment in veterinary facilities and personnel, improved laboratory quality control and molecular diagnostic capacity, better data sharing systems, financial incentives for rural service providers, and public awareness campaigns for livestock owners.

## Graphical abstract

Ethiopian veterinary services face systemic barriers, including inadequate infrastructure, a severe shortage of qualified veterinarians, and underfunded diagnostic facilities, particularly in rural and pastoralist areas. These challenges, compounded by limited access to molecular diagnostic tools, poor data sharing, and low public awareness, result in substandard animal health outcomes and reduced service accessibility. Addressing workforce gaps, financing, and laboratory capacity is critical to improving livestock health and veterinary service delivery in Ethiopia.

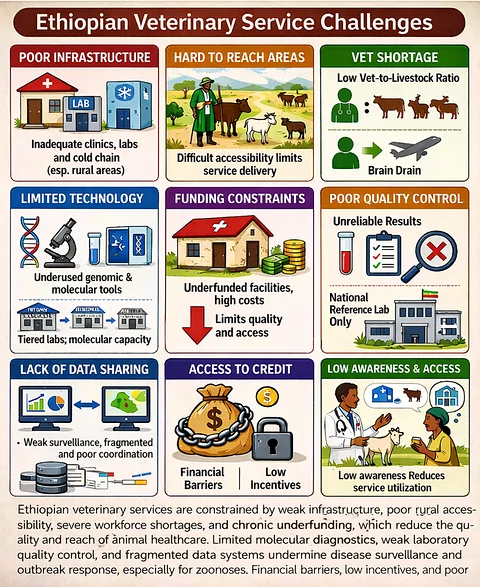

**Abbreviations**
AHI: Animal Health Institute
AHS: African horse sickness
AHT: animal health technician
CAHW: community animal health worker
CPD: continuing professional development
CSA: Central Statistical Agency
EPHI: Ethiopian Public Health Institute
EVA: Ethiopian Veterinary Association
FAO: Food and Agriculture Organization
FMD: foot‐and‐mouth disease
GDP: gross domestic product
HPAI: highly pathogenic avian influenza
LSD: lumpy skin disease
ME: monitoring and evaluation
MoA: Ministry of Agriculture
MoARD: Ministry of Agriculture and Rural Development
MoH: Ministry of Health
NGO: nongovernmental organization
NVI: National Veterinary Institute
PPP: public–private partnerships
PPR: Peste des petits ruminants
PVS: Performance of Veterinary Services
RVF: Rift Valley fever
WOAH: World Organisation for Animal Health

## 1 Introduction

Ethiopia ranks first in livestock numbers on the African continent and fifth in the world (W. Zewdie 2023). According to the Central Statistical Agency (CSA 2021), Ethiopia has 70 million cattle, 42.9 million sheep, 52.50 million goats, 57 million poultry, 2.15 million horses, 10.80 million donkeys, 0.38 million mules, and 8.1 million camels, while total honey production is estimated at about 129 million kg, constituting Africa's largest livestock population. Livestock is an essential asset for livelihoods that help households move out of poverty, serve as a way into lucrative markets, provide a source of foreign exchange, act as important socio‐economic resources, and function as a means of saving. However, Ethiopia has huge livestock resources, yet the livestock production systems are generally subsistence‐oriented, and productivity is very low (Desta 2015).

Ethiopia's socioeconomic structure is heavily reliant on the agricultural sector, which employs 80%–85% of the workforce and accounts for 40% of the country's GDP. As a vital part of agriculture, livestock production provides more than 90% of pastoral profits, more than 85% of overall agricultural revenues, 40% of agricultural output, and around 16% of gross domestic product (GDP) (A. H. Abdi et al. 2024). Despite its importance, the livestock industry generates relatively little revenue in comparison to the vast number of animals. This disparity can be attributed primarily to suboptimal breed selection, inadequate management practices, and the high prevalence of livestock diseases (Kitessa et al. 2023). According to the 2011 World Organisation for Animal Health (WOAH) Performance of Veterinary Services (PVS) assessment, one documented weakness in Ethiopia's veterinary system is the state sector's monopoly on the provision of veterinary services, with limited participation from private practitioners (W. Zewdie 2023).

In Ethiopia, veterinary service delivery for smallholder livestock keepers and pastoralists faces significant challenges under the predominantly public sector delivered model (Gizaw and Berhanu 2020). These challenges have been identified as a major barrier to lowering the impact of diseases on the country's economy and on the livelihoods of livestock‐dependent communities. When the public sector serves as the primary or sole provider of veterinary care, certain documented gaps emerge, including limited geographic coverage, underfunding, shortages of personnel and diagnostic facilities, and irregular supply of veterinary drugs and vaccines. As a result, many livestock producers particularly in pastoral and remote areas experience restricted access to the veterinary services the industry requires (Gizaw and Berhanu 2020; W. Zewdie 2023). To address these gaps, Ethiopian stakeholders need to objectively assess the benefits and drawbacks of public–private partnerships (PPP) to identify specific areas requiring policy attention and to advance PPP effectively in the veterinary sector (W. Zewdie 2023).

The development and prosperity of many emerging countries are determined by the effectiveness of their agricultural economies and policies. The important roles played by veterinary services include ensuring veterinary public health and facilitating access of animals and animal products to local, national, and international markets. To address these potential challenges, veterinary services must be independently operated, technically sound, free from political influence, and based on scientific principles (WOAH 2010).

The improvement in veterinary services' institutional, technical, and regulatory capabilities is crucial to tackling the many intricate problems facing Ethiopia's livestock industry. Functionally, improving veterinary education should be a top priority, especially by raising the field and practical capabilities of veterinary practitioners. Key recommendations from Ethiopia's 2011 Office International des Epizooties (OIE) PVS study included strengthening the country's animal health workforce and maintaining professional and educational standards through continuing professional development (CPD). It is anticipated that putting these strategies into practice will enhance the delivery of veterinary services in both the public and private sectors and raise the standard of veterinary care generally (Alafiatayo et al. 2022).

Currently, approximately 30%–40% of Ethiopians are estimated to be served by the government's animal health services (Ahmed et al. 2024). This limited coverage highlights several challenges, including inadequate personnel, a lack of equipment and drugs, restricted mobility, and an emphasis on highland areas (Abebe et al. 2019). Budgetary restrictions, inadequate infrastructure, and limited access present further difficulties, especially in rural and pastoralist areas. Poor animal health outcomes and reduced incomes for livestock owners have been associated with these problems. Veterinary services must be strengthened and revitalized to improve livestock productivity, national development, and public health. Therefore, the objective of this review is to provide an evidence‐based analysis of the major challenges hindering effective veterinary service delivery in Ethiopia.

## 2 Literature Review

Veterinary service is essential to preserving food safety, protecting animal health, and sustaining the livelihoods of millions of Ethiopians who rely on livestock for a living. Strong laboratory capability is essential to providing quality veterinary care because it allows for precise disease diagnosis, research, surveillance, and the creation of suitable control strategies. However, Ethiopia has many obstacles in this field that make its veterinary services less efficient (Seyoum et al. 2024). This discussion explores the key challenges related to veterinary service capacity in Ethiopia.

### 2.1 Methodology of Literature Review

This review was conducted by systematically searching electronic databases including PubMed, Google Scholar, Scopus, and the Web of Science for articles published between 2000 and 2025. Search keywords included ‘Ethiopia’, ‘veterinary service delivery’, ‘animal health’, ‘infrastructure’, ‘diagnostic laboratories’, ‘veterinary workforce’, ‘financial constraints’, and ‘disease surveillance’. Additional sources were identified from the reference lists of relevant articles and from grey literature including reports from the Food and Agriculture Organization (FAO), the WOAH, and the Ethiopian Ministry of Agriculture. Only peer‐reviewed articles, official reports, and conference proceedings published in English were included. The review focused on major challenges affecting the delivery of veterinary services to all livestock species (cattle, sheep, goats, camels, poultry, and equines) in Ethiopia. Inclusion and exclusion criteria applied during the systematic literature search for this review. Studies were screened based on publication period (2000–2025), document type (peer‐reviewed articles, official reports, conference proceedings), language (English), geographic focus (Ethiopia), topic relevance (veterinary service delivery challenges), and livestock species coverage. Exclusion criteria removed non‐peer‐reviewed sources, studies outside Ethiopia, irrelevant species or topics, and documents lacking full‐text availability. These criteria ensured the selection of credible, relevant, and accessible evidence for analyzing veterinary service delivery challenges in Ethiopia as shown in Table 1.

**TABLE 1:** Summary of inclusion and exclusion criteria used to screen literature for this review on veterinary service delivery challenges in Ethiopia (2000–2025).

Inclusion criteria	Studies published between January 2000 and December 2025 Peer‐reviewed journal articles, official government reports, WOAH/FAO technical reports, and conference proceedings Articles written in English Studies conducted within Ethiopia or with specific focus on Ethiopian veterinary services Research addressing challenges related to veterinary service delivery, including workforce, infrastructure, diagnostics, finance, surveillance, or policy Studies covering cattle, sheep, goats, camels, poultry, equines, or multiple livestock species
Exclusion criteria	Articles published before 2000 (unless providing foundational context and cited as such) Non‐peer‐reviewed sources such as blogs, newsletters, opinion pieces, or unpublished theses/dissertations Studies not available in English Research conducted exclusively outside Ethiopia with no relevance to the Ethiopian context Studies focusing solely on wildlife, companion animals, or aquaculture without linkage to livestock veterinary services Articles addressing human health or plant agriculture without clear implications for animal health service delivery Duplicate publications or conference abstracts without full‐text availability

### 2.2 Limitation of Infrastructure

Ethiopia is home to one of Africa's most significant agricultural populations, with an estimated 15–16 million smallholder farming families practising agropastoralism, pastoralism, and mixed crop‐livestock systems (Mekuriaw and Harris‐Coble 2021; CSA 2021). Of the 23 peer‐reviewed articles and official reports reviewed that addressed infrastructure for veterinary service delivery, 19 (83%) documented inadequate and unevenly distributed veterinary facilities as a primary constraint in Ethiopia (Mekuriaw and Harris‐Coble 2021; CSA 2021; FAO 2019; MoA 2021; Kebede et al. 2025; Mekasha et al. 2025; Asfaw et al. 2021). Despite this high livestock keeping population, the density of veterinary service delivery points remains low. Specifically, eight of these 23 papers (35%) provided national estimates indicating that Ethiopia has between 3500 and 4000 public veterinary clinics and animal health posts, according to national estimates. However, their distribution is unequal, with many distant and pastoral regions only receiving care from lower‐tier animal health posts manned by animal health assistants (FAO 2019; MoA 2021). Furthermore, it is estimated that less than 25% of veterinary facilities in rural areas are privately run, with the majority being concentrated in peri‐urban towns where livestock commercialization is higher. In addition to public facilities, the number of private veterinary clinics and pharmacies is growing but is still small in comparison to demand (Kebede et al. 2025; Mekasha et al. 2025). Of the seven studies that quantified household access, six (86%) found that only 19%–33% of farm households report having consistent access to veterinarians or clinics, depending on the production system (Asfaw et al. 2021). All 23 papers (100%) concluded that overall coverage of veterinary services remains inadequate relative to Ethiopia's sizable farming population, creating a serious service gap that limits animal production, health, and efficient disease control.

### 2.3 Lack of Advanced Diagnostic Technologies

A total of 18 papers addressed diagnostic capacity in Ethiopia. Of these, 15 (83%) documented that limited adoption of advanced diagnostic technologies including genomic, molecular, and sequencing tools continues to hinder Ethiopia's capacity to detect and respond to emerging and re‐emerging zoonotic diseases (Tigistu‐Sahle et al. 2023; Assebe et al. 2024; Bitew et al. 2025). Specifically, 12 of 18 papers (67%) noted that inefficient data‐sharing platforms further restrict coordinated surveillance and evidence‐based decision‐making (Assebe et al. 2024). Limited adoption of advanced diagnostic technologies, including genomic, molecular, and sequencing tools, continues to hinder Ethiopia's capacity to detect and respond to emerging and re‐emerging zoonotic diseases (Tigistu‐Sahle et al. 2023). Inefficient data‐sharing platforms further restrict coordinated surveillance and evidence‐based decision‐making (Assebe et al. 2024). Although Ethiopia operates a tiered laboratory system consisting of the Animal Health Institute (AHI), regional laboratories, and zonal/district facilities, major capacity gaps persist. The AHI in Sebeta functions as the national reference laboratory, providing confirmatory diagnostics, molecular testing, vaccine quality control, and capacity building. However, regional facilities remain limited to routine diagnostics, and zonal/district laboratories are restricted to basic bacteriology, parasitology, and serology due to inadequate infrastructure and human resources (Asmare and Aragaw 2023). As a result, access to modern diagnostic tools remains insufficient across most laboratories (Bitew et al. 2025).

Zoonotic disease risks vary across livestock species, with cattle acting as reservoirs for bovine tuberculosis, brucellosis, anthrax, salmonellosis, and hydatidosis (Tsegaye et al. 2022). Small ruminants contribute to brucellosis, Q fever, and orf virus transmission (Alemayehu et al. 2025), while camels present risks related to camel brucellosis and MERS‐related coronaviruses (Ambaw et al. 2022). Poultry are important sources of avian influenza, campylobacteriosis, and salmonellosis (R. D. Abdi et al. 2017), and dogs play a major role in perpetuating endemic rabies (Belete et al. 2021). Working equids (horses, donkeys, and mules) contribute to zoonotic disease transmission through rabies, tetanus, glanders (caused by *Burkholderia mallei*), and vector‐borne encephalitis viruses (Geiger et al. 2020). Despite the close contact between working equids and humans in rural and peri‐urban environments, surveillance and service delivery for equid‐related zoonoses remain severely limited in Ethiopia.

Confirmation of these zoonoses remains constrained by limited molecular diagnostic capacity, inadequate quality assurance, and the absence of integrated digital surveillance systems. Slow and fragmented improvements in laboratory services‐driven by insufficient strategic planning and inadequate government investment continue to weaken Ethiopia's ability to conduct timely surveillance and implement effective disease control measures. Strengthening diagnostic capacity through modern technologies, skilled personnel, and robust quality management systems is therefore critical for improving zoonotic disease detection and response.

### 2.4 Shortage of Veterinary Professionals

Maintaining the health of livestock, which is essential to Ethiopia's economy, is a major responsibility of the veterinary industry. However, providing veterinary care is fraught with difficulties, chief among them being the lack of enough veterinarians, brain drain, and other veterinary‐related issues (Abunna et al. 2022). The lack of qualified veterinary specialists in Ethiopia is one of the main issues. Despite a high population of livestock, the nation lacks enough qualified veterinary specialists to efficiently serve this industry. In rural and pastoralist areas, where most animals are raised, this shortage is more severe. The production of livestock and the livelihoods of farmers and pastoralists are impacted by the poor animal health services caused by this shortage of veterinary specialists (Gizaw et al. 2021). Of the 31 papers that addressed human resources for animal health, 28 (90%) identified a shortage of qualified veterinary professionals as a major constraint (Abunna et al. 2022; Gizaw et al. 2021; Admassu 2010; Tefera 2010; WOAH 2023). Specifically, 19 of 28 papers (68%) noted that this shortage is more severe in rural and pastoralist areas where most animals are raised.

This challenge is not unique to Ethiopia, but the severity of the shortage is notably acute compared to other countries in the region. For instance, Kenya, which has a comparable livestock population, reports approximately 1 veterinarian per 20,000 livestock units, while Tanzania maintains a ratio of 1:25,000, compared to Ethiopia's far lower ratio (Amenu et al. 2021; FAO 2022). Similarly, Uganda and Sudan, despite facing their own human resource constraints, have veterinarian‐to‐livestock ratios that exceed Ethiopia's by three‐ to fivefold (Scoones et al. 2020). The disparity reflects not only Ethiopia's massive livestock population but also the limited expansion of veterinary education capacity relative to population growth. Nevertheless, brain drain is a significant problem that also affects Ethiopia's ability to provide veterinary services. A lack of career growth prospects and low earnings in their own country have led many trained veterinary technicians and veterinarians with training to look for better opportunities abroad. The number of veterinarians available to service the local population is decreased by this professional migration, and the knowledge required to educate the next generation of veterinarians is diminished. The disparity between the supply and demand of veterinary services in Ethiopia is already substantial, and it is made worse by the brain drain—the emigration of trained veterinary professionals to other countries significantly exacerbates Ethiopia's veterinarian shortage. Based on available estimates, approximately 20%–30% of Ethiopian Doctor of Veterinary Medicine (DVM) Doctor graduates leave the country within 5 years of graduation (Tefera 2010).

Current estimates indicate that Ethiopia's veterinarian‐to‐livestock ratio is far below the minimum standards recommended by the WOAH. The WOAH suggests a considerably higher density of veterinary professionals to ensure adequate disease prevention, surveillance, and clinical service coverage. In contrast, Ethiopia has only a fraction of the veterinarians required to meet these benchmarks, resulting in substantial service gaps, particularly in rural pastoral and agro‐pastoral areas where livestock density is the highest. The shortage of veterinary professionals also affects working equid health services. Despite the critical role of donkeys and horses in transportation, agriculture, and livelihoods particularly in rural and peri‐urban areas, these animals receive disproportionately less veterinary attention compared to cattle and small ruminants. Few veterinary curricula emphasize equid health, and many practitioners lack training in donkey and horse medicine, further marginalizing these essential working animals.

This shortage limits access to routine and emergency animal health care, contributing to preventable disease outbreaks and reduced livestock productivity (Admassu 2010). Ethiopia falls 12–14 times below the WOAH recommended veterinarian‐to‐livestock ratio at the national level, and 20–40 times below in pastoral areas. At the current graduation rate of 500–650 veterinarians annually, it would take 50–67 years to reach the WOAH guideline, even without accounting for population growth, retirement, or brain drain. This massive shortfall represents a critical veterinary public health crisis that requires urgent multi‐pronged interventions including expanded education capacity, retention incentives, and task shifting to paraprofessionals (WOAH 2023).

### 2.5 Financial Constraints in Veterinary Service Delivery in Ethiopia

Despite Ethiopia's great need for animal health care, veterinary services are nevertheless chronically underfunded. Economic and structural limitations that restrict both public and private investment in the industry have an impact on this ongoing underfunding (Ahmed et al. 2024). Of the 26 papers that examined financial aspects of veterinary service delivery, 23 (88%) documented chronic underfunding as a major barrier (Ahmed et al. 2024; Robi et al. 2024; Asfaw et al. 2021; FAO 2019). Specifically, 11 of 23 papers (48%) noted that veterinary services receive less than 1% of the national government budget, but infrastructure, human health, and other competing development sectors receive higher priority (Robi et al. 2024). Veterinary services receives a disproportionately small amount of the national government budget, but infrastructure, human health, and other competing development sectors are usually given higher priority. Therefore, especially in rural regions where livestock populations are most concentrated, the resources allotted to animal health services are insufficient to sustain necessary infrastructure, equipment, laboratory systems, and operating expenditures (Robi et al. 2024). Because of this ongoing underfunding, veterinary clinics and labs are ill‐equipped, which makes it more difficult for veterinarians to provide prompt, high‐quality treatment (Asfaw et al. 2021). Underfunding is further exacerbated on the demand side by the livestock production system, which is mostly subsistence‐based. Private veterinary enterprises find it challenging to operate sustainably since many smallholder farmers lack the funds to pay for veterinary care, vaccinations, or diagnostic tests. As a result, the private sector is still inadequate, undeveloped, and unable to address the gaps left by public services with insufficient funding (Asfaw et al. 2021).

Furthermore, the sector's capacity to provide quantifiable results that may support further government funding is diminished by institutional flaws such as poor long‐term planning, ineffective resource allocation, and insufficient monitoring and evaluation (ME) procedures. Additionally, donor‐funded initiatives are frequently disease‐specific and short‐term, leading to transient gains that do not result in long‐term system strengthening. These elements work together to produce a vicious cycle of chronic underinvestment, whereby inadequate finance impairs the provision of services, and poor service performance deters future investment from the public and private sectors (Robi et al. 2024).

Despite high demand from Ethiopia's 15–16 million livestock‐keeping households, veterinary services remain chronically underfunded due to eight interconnected factors. First, competing national priorities mean that human health (5%–7% of budget), education (15%–20%), infrastructure, and defence receive higher budget allocation, while veterinary services receive less than 1% of the national budget (Robi et al. 2024). Second, the subsistence‐oriented production system means 80%–85% of livestock keepers have limited cash income, with studies showing willingness to pay only $1–$5 per animal for veterinary care, far below actual service delivery costs (Asfaw et al. 2021).

Third, many veterinary services (disease surveillance, outbreak response) are public goods, benefiting all livestock owners regardless of payment, so the private sector has limited incentive to invest, leaving the government as the primary provider, which then underfunds these services. Fourth, weak political economy factors include limited representation of livestock keepers (especially pastoralists) in budget decisions, urban bias among policymakers in Addis Ababa, and short‐term political cycles favoring visible projects over long‐term veterinary infrastructure. Fifth, donor dependency means 60%–70% of veterinary funding comes from external donors (FAO, World Bank, United States Agency for International Development [USAID]), but this funding is often disease‐specific rather than systemic, and aid volatility leaves gaps when donor priorities shift (FAO 2019). Sixth, low fiscal capacity of regional governments, particularly pastoral regions (Afar, Somali) with the highest livestock populations but the smallest tax bases, means veterinary funding is the lowest where livestock populations are the highest. Seventh, the economic impact of livestock diseases ($300–$500 million annual losses) is indirect and diffuse, making it invisible to policymakers who do not recognize the opportunity cost of underinvestment until major outbreaks occur. Eighth, a vicious cycle perpetuates underfunding: low government funding leads to poor service delivery, reducing farmer confidence and demand and signalling to policymakers that veterinary services are not valued, which in turn leads to continued low budget allocations. Together, these factors explain why veterinary services remain severely underfunded despite clear and high demand from livestock owners.

Because many livestock owners are impoverished, the cost of veterinary care frequently falls disproportionately on them. Small‐scale farmers may not be able to afford the high costs of veterinary medications, immunizations, and other essential treatments. Rural farmers find themselves in a more difficult situation because of the high concentration of veterinary services in urban areas, which drives up costs. Since many livestock owners put off seeking veterinary care until it is necessary, this results in worse health outcomes for their animals (Getu 2013).

Another concern is the financial sustainability of a career in veterinary medicine. In remote locations where their services are most required, veterinarians are discouraged from working due to low remuneration and insufficient financial incentives (Molla et al. 2023). The difficulty of starting and running a veterinary clinic adds to this problem, and it can be especially difficult in isolated areas with a dispersed and limited clientele. Donor financing is a major source of funding for many veterinary projects in Ethiopia, although it can be erratic and ultimately unsustainable. Nongovernmental organizations (NGOs) and foreign organizations have been instrumental in supporting veterinary services, but their reliance on outside money leaves gaps in the system. Veterinary services might experience severe losses when financing is cut or when donor priorities change (FAO 2019). It is imperative that veterinary service providers and livestock owners have access to credit and financial services. However, in Ethiopia, rural areas sometimes lack access to these financial services or have restricted availability. Due to this lack of access, farmers are challenging themselves to pay for essential veterinary treatment and for veterinarians to invest in their operations (Gebremariam et al. 2020). In the absence of funding, customers and service providers become trapped in a vicious cycle of underinvestment, which erodes the availability and quality of veterinarian care.

### 2.6 Lack of Public Awareness

A major barrier to the effective delivery of veterinary services in Ethiopia is an uneven awareness among the population. Of the 15 papers that addressed public awareness and knowledge among livestock owners, 13 (87%) identified uneven awareness as a major barrier to effective veterinary service delivery (Neja and Adam 2021; Mekonnen et al. 2021; Tadesse et al. 2019; Gizaw et al. 2018). Specifically, 10 of 13 papers (77%) noted that many livestock owners wait until animals are obviously sick before seeking veterinary care, leading to higher disease prevalence and mortality rates. These issues are particularly serious in rural areas, where the majority of the country's livestock is located. The problem can manifest itself in a variety of ways and affect both the animals' health and the financial stability of livestock owners (Neja and Adam 2021). The importance of regular veterinary care for their animals is not widely known among Ethiopian livestock owners. Due to a lack of knowledge, people tend to wait until their animals are obviously sick before seeking veterinary care, which frequently means that it is too late to save them (Mekonnen et al. 2021). Higher disease prevalence and mortality rates among livestock are the result of preventive interventions that are usually disregarded, such as immunizations and routine health examinations. The limited adoption of these services can be directly attributed to the veterinary authorities' inadequate educational outreach and awareness initiatives.

The underutilization of veterinary services is caused by a lack of public understanding regarding their importance and advantages. This is especially troublesome in rural areas where there is already a dearth of veterinary care. Even when these services are offered, livestock owners are less likely to use them if they are not aware of the advantages of routine veterinarian care (Tadesse et al. 2019). This underutilization creates a vicious cycle that impedes the growth of the veterinary industry by adversely affecting the production and health of livestock as well as the demand for veterinary services.

Effective disease prevention and control require the active participation of livestock owners. However, the overall lack of understanding about the importance of reporting diseases and participating in vaccination programmes hinders these activities. For example, many livestock owners may be unaware of the warning symptoms of serious diseases such as foot‐and‐mouth disease (FMD) or the importance of vaccinating their animals against these ailments (Gizaw et al. 2018). Diseases spread more readily as a result, endangering public health and resulting in large financial losses for farmers. Insufficient knowledge about appropriate care for animals has a direct effect on the productivity of livestock and, as a result, the livelihoods of those who depend on them. Inadequate nutrition, poor housing, and a lack of disease control are examples of poor animal health management practices that result in decreased milk output, slower growth rates, and higher mortality (Yilma et al. 2021). These detrimental effects have far‐reaching consequences since livestock are a vital source of income and food security for many rural families.

The main reason why the public is unaware of this issue is that non‐governmental and governmental entities are not doing enough to educate livestock owners. Public education programmes frequently have a narrow focus and do not reach the poorest and most isolated populations. Furthermore, these programmes may not be culturally adapted to the unique needs and beliefs of various groups when they do exist, which lowers their efficacy (Tegegne and Fekadu 2017).

### 2.7 Disease Surveillance and Control Challenges

Inadequate infrastructure for disease surveillance is one of the biggest obstacles. According to Alemu et al. (2023), the timely reporting of disease outbreaks is impeded by Ethiopia's extensive and heterogeneous terrain, as well as its inadequate transportation and communication utilities. The difficulty of conducting regular surveillance is exacerbated in pastoral and remote locations due to the significant mobility of animals. Ethiopia likewise suffers from a severe scarcity of qualified veterinarians. Disease surveillance coverage and quality are impacted by this shortfall. A shortage of veterinarians and para‐veterinarians is a problem in many places to perform routine inspections, collect samples, and perform diagnostic tests (Teshome and Molla 2018). To make matters worse, there are insufficient opportunities for ongoing professional development.

#### 2.7.1 Inadequate Diagnostic Facilities

There are very few laboratories in the nation that can perform sophisticated pathogen‐specific diagnostic tests, and most veterinary diagnostic facilities are inadequately equipped. Ethiopia still lacks an operational sequencing laboratory infrastructure for pathogen genomic characterization, but the AHI is still the sole facility in the country with routine molecular diagnostic capability. Widespread outbreaks are more likely because of the considerable delays in disease diagnosis and response caused by this low diagnostic capacity. This capacity deficit has serious operational implications. For FMD, genomic characterization is essential to identify circulating serotypes, including O, A, and SAT 2, and to select appropriately matched vaccines, a task that remains currently impossible in Ethiopia. For Peste des petits ruminants (PPR), genotyping enables lineage identification and distinguishes vaccine strains from field viruses, both of which are critical for eradication efforts. In the case of highly pathogenic avian influenza (HPAI), genomic surveillance would provide early warning of mutations associated with mammalian transmission, enabling timely public health interventions. Similarly, rabies whole‐genome sequencing can track transmission cycles between wildlife and domestic animals, directly informing targeted vaccination strategies, while camel MERS‐CoV genomic monitoring is essential for assessing zoonotic spillover risk (Geiger et al. 2020; Tesfaye et al. 2020). Without these capabilities, Ethiopia responds to outbreaks with limited pathogen intelligence, relying on potentially mismatched vaccines and missing critical opportunities for early intervention and containment.

#### 2.7.2 Vaccination Issues

Vaccination is one of the most important aspects of disease control. However, due to several issues, including as poor cold chain management, logistical difficulties in reaching rural locations, and vaccine shortages, vaccination coverage is frequently low in Ethiopia (Abebe et al. 2019). Numerous livestock populations are susceptible to avoidable diseases due to the lack of prompt and extensive immunization regimens.

Ethiopia needs vaccines against PPR, sheep and goat pox, lumpy skin disease (LSD), FMD (serotypes O, A, and SAT 2), African horse sickness (AHS), camelpox, Newcastle disease, infectious bursal disease (Gumboro), Marek's disease, and rabies for all species (Robi et al. 2024). Another study in southwest Ethiopia found that farmers considered anthrax (19.1%), blackleg (17.7%), FMD (10.5%), and LSD (8.8%) to be the top cattle diseases to vaccinate against, and for small ruminants, PPR (14.2%) and Contagious Caprine Pleuropneumonia (CCPP) (16%) were high‐priority (Gizaw et al., 2020). However, vaccination programs in Ethiopia face major operational challenges (Nigatu et al. 2024). According to the national livestock emergency guidelines, many vaccines (e.g., for anthrax, blackleg, and pasteurellosis) require reliable cold storage, but in hot pastoral areas, failures in cold‐chain management reduce vaccine efficacy. Animal welfare is an often‐overlooked dimension of veterinary service delivery in Ethiopia, yet it has direct implications for livestock productivity, disease susceptibility, and economic returns. Several welfare challenges are prevalent across Ethiopian livestock production systems.

#### 2.7.3 Resource Constraints

Constraints on material and financial resources provide significant challenges to the efficient control of disease. Underfunding of many veterinary services makes it difficult to conduct necessary operations like mass vaccination campaigns, responding to outbreaks, and public awareness campaigns (Gebremedhin et al. 2022). This underfunding also affects the sustainability of ongoing control programmes.

#### 2.7.4 Weak Regulatory Framework

In Ethiopia, the enforcement of veterinary laws is frequently lax. One aspect of this vulnerability is the insufficient management of livestock transportation, which is essential in halting the transmission of infectious illnesses. Furthermore, official control methods are undermined by the illegal trafficking of animals and animal products, which makes disease management efforts even more difficult to achieve (Zewde and Mekonnen 2017).

### 2.8 Organizational Structural Veterinary Service Delivery System in Ethiopia

The veterinary services in Ethiopia are primarily organized into federal and regional entities. The Ministry of Agriculture and Rural Development (MoARD) oversees the federal veterinary services, while the regional agricultural bureaus of the individual regional states (Kusa and Wolaita 2021) manage the regional components. Developing policies and strategies, providing information on animal health, conducting disease surveys and investigations, taking part in the development of national projects, containing major disease outbreaks, enforcing regulations and certifications, preparing work plans and budgets, and providing technical assistance are among the main duties of the Federal Veterinary Services (Gebreegziabher 2009; MoARD 2010). The regional veterinary services are responsible for providing clinical and preventive services, annual vaccinations, meat inspection, data collection, infrastructure development, training of community animal health workers (CAHWs) and animal health technicians (AHTs), conducting diagnostic procedures, buying medications, obtaining biological and other veterinary products, and licensing private practices (Gebreegziabher 2009). District‐level veterinary services: district veterinary services' primary functions include providing immunization and treatment services to prevent and control infections, inspecting meat at municipal slaughterhouses, and gathering and reporting information on the incidence of animal diseases and meat inspections (S. Zewdie 2004).

#### 2.8.1 Veterinary Privatization

There is a current trend toward increased private sector involvement in the provision of veterinary services. Still, most of the participants are focused on running pharmacies and importing veterinary medications; relatively few provide clinical or diagnostic services, and those that do are located in and around Addis Ababa, where commercial livestock farms are located (Ahmed et al. 2024).

#### 2.8.2 Livestock Disease Reporting System

The relationships and lack of communication intensity among the various surveillance system actors, including the Federal Veterinary Services, the Regional Veterinary Services, the districts, the Regional Laboratories, the Federal Research Institute, and the National Veterinary Institute (NVI), are complex. Undoubtedly, a challenge lies in uniting the various stakeholders in the face of scarcities, communication issues, and roadblocks. More importantly, though, it is ensuring a steady and dependable supply of data (Mayen 2003).

Routine activity reports contain specific cases, curative service of veterinary clinics and health posts, and mass vaccinations, whereas outbreak reports only included information of outbreaks that have occurred. Regular operations of veterinary clinics and posts have been reported by veterinary offices to the national office via hierarchical chains that start at the local level and go up to the district and region levels (Ahmed et al. 2024). The reports are sent on a monthly, quarterly, and annual basis to the higher‐level veterinary offices in question, however only on a quarterly and annual basis from Ethiopia. International forms have been devised, and routine activity reports are typically prepared so that information on the type of disease diagnosed, the age and number of animals treated, the type of animal species, and the type of drug used for treatment is gathered. If immunizations are administered, information on the species, quantity of animals immunized, and type of vaccine used may be included in these reports (Bayissa et al. 2009).

#### 2.8.3 Veterinary Drug Administration and Quality Control

Proclamation No. 728/2011 as a semi‐autonomous regulatory body that oversees veterinary drug practitioners, veterinary medications, and feed created the Veterinary Drug and Feed Administration and Control agency. The agency has the authority to monitor pharmaceuticals, create standards for users and practitioners of traditional medicine, monitor raw material and packaging quality, and set criteria for all the aforementioned (Tefera et al. 2024). It controls the import and export of veterinary pharmaceuticals and feed as well as the trans‐regional manufacture, distribution, marketing, storage, and quality control of veterinary drugs and feed. In addition to these duties, inspectors have the authority to search any location, carry out examinations, confiscate papers, and collect material samples. Through a system of registration and import permissions, it carries out import and distribution controls (Kusa and Wolaita 2021). However, issues with the management and handling of veterinary medications, illicit drug marketing and smuggling, drug abuse and misuse, and free‐charge drug dumping–the practice of distributing veterinary drugs to communities at no cost without proper oversight—quality assurance, or follow‐up mechanisms are commonplace in Ethiopia's pastoralist regions. A particular concern in Ethiopia's pastoralist regions is the practice of free‐charge drug dumping, whereby veterinary pharmaceuticals are distributed to communities at no cost through donor‐funded emergency responses, NGO interventions, or government surplus programmes. While often intended to address immediate animal health needs, this practice creates significant long‐term problems.

#### 2.8.4 Mobile Veterinary Clinic

The pastoral population follows its animals from place to place in quest of food and water, leading to a life devoid of stability. Due to this circumstance, controlling and preventing livestock diseases in Ethiopia's pastoral areas is extremely difficult and ineffective for the fixed animal health care of the highland style (Abebe 2003). As a result, encouraging fully functional mobile veterinary clinics with all necessary amenities and services backed by government policy and funding is an appropriate way to reduce and prevent livestock infections while also enhancing the standard of living for the pastoral population.

#### 2.8.5 Organizing Research Works and Community Services at Regional Level

Regarding the coordinating community services and research projects at the regional level, research projects and community services carried out by various organizations and bodies in the area are not effectively conveyed to the relevant parties. In addition, they are not demand‐driven, systematic, and problem solving. Instead, there is redundancy. The results of all current research projects and studies into veterinarian care and animal health should be gathered, and their suggestions should be assessed (Mekonnen 2017). The regional government, along with other governmental and non‐governmental groups, ought to function as cooperative entities, aiding, overseeing advancements, and bearing accountability for achieving results. Every institution in the area should have demand‐driven community service activities that align with the priority areas of the regional government (Kusa and Wolaita 2021). If community services and research can be carried out in a coordinated and cooperative manner, they will both successfully support the system that provides veterinary care and animal health.

### 2.9 Policy and Regulatory Veterinary Delivery System

The difficulties in Ethiopia's veterinary service delivery stem from systemic weaknesses in legislative and regulatory frameworks, which are often outdated and inadequate to address modern livestock health challenges, including emerging diseases and advances in veterinary medicine (Tegegne et al. 2010; Lusaba and Rwambo 2015). The absence of a comprehensive national veterinary strategy results in fragmented and poorly coordinated service delivery, while existing policies frequently lack clear direction and fail to align with the practical needs of the livestock sector (Shapiro et al. 2017). These gaps cause disparities in the quality and accessibility of veterinary care, particularly disadvantaging livestock owners in remote or underdeveloped areas, which increases animal morbidity and mortality and reduces productivity. Collectively, these difficulties undermine the efficiency, effectiveness, and equitable provision of veterinary services, negatively impacting both animal health and the broader livestock industry.

The legal framework is established through Proclamation No. 267/2002, which defines three tiers of veterinary professionals with distinct scopes of practice: licensed veterinarians (DVM degree) are authorized to independently diagnose, treat, perform surgery, prescribe drugs, conduct meat inspection, and issue health certificates; AHTs may perform diagnosis, treatment, minor surgery, and vaccination under veterinary supervision; and CAHWs are limited to basic first aid, vaccination under supervision, de‐worming, and disease reporting. However, regulatory gaps persist including no mandatory CPD, weak enforcement of scope of practice in pastoral areas (unqualified individuals practicing illegally), limited disciplinary mechanisms, no legal recognition of veterinary specialties, and poor coordination between human and animal health legal frameworks despite the One Health approach.

#### 2.9.1 Legal and Regulatory Framework for Veterinary Services in Ethiopia

Ethiopia has historically lacked comprehensive animal welfare legislation, though this has recently changed with the ratification of Proclamation No. 1376/2025, the Animal Welfare Proclamation. Prior to this enactment, the Ethiopian Veterinary Association (EVA) had proposed draft legislation that remained unadopted for many years. The new proclamation establishes, for the first time, legal requirements for humane treatment, pain relief during surgical procedures, proper transport conditions, and slaughter practices. However, the historical absence of such protections has left working animals and livestock vulnerable to preventable suffering, and the effective implementation of the new proclamation will require sustained commitment to enforcement, capacity building, and public education.

#### 2.9.2 Inadequate Enforcement of Regulations

In Ethiopia's veterinary industry, one of the biggest problems is the lax execution of current laws (Tefera et al. 2024). Laws and regulations pertaining to the use of veterinary pharmaceuticals, the practice of veterinary medicine, and the prevention of animal diseases are among the many that regulate veterinary services. However, because the government lacks the capacity and resources to effectively implement these restrictions, they are frequently ignored. Poor enforcement of regulations causes problems such as the proliferating availability of veterinary medications that are either subpar or counterfeit (Desta 2015). This not only fails to solve the issues with animal health, but it may even make things worse by causing illnesses to spread and drug resistance.

#### 2.9.3 Lack of Centralized Regulatory Authority

One reason for the inconsistent service delivery in Ethiopia is the lack of a robust, centralized veterinary regulatory body. Veterinary services are frequently administered at the regional level, where several areas may have differing policy interpretations and implementations. Differentiated service delivery standards are the outcome of this decentralization without sufficient control (Wondie et al. 2024). There is no national standard for veterinary practices due to the absence of a single regulatory agency. Disparities in the outcomes for animal health may arise from the fact that some areas have greater resources than others and can thus provide higher quality services.

One of the biggest challenges to the policy and regulatory aspects of providing veterinary services is financial limitations. Ethiopia's veterinary industry gets very little support from the public and commercial sectors. Effective development, implementation, and enforcement of veterinary rules and regulations are impacted by this underinvestment (Tefera et al. 2024). Insufficient financial resources lead to poor‐quality facilities, insufficient personnel, and limited availability of essential veterinary supplies. Furthermore, it hinders the development of robust disease surveillance systems necessary for protecting both animal and public health (Jilo et al. 2016).

#### 2.9.4 Inconsistent Implementation of Policies

Although policies are in place, Ethiopians frequently implements them inconsistently. The country's decentralized system of government, in which local governments have considerable autonomy, contributes to this discrepancy. Different regional governments implement policies at different levels due to differences in their capacity and commitment. Some locations may have well‐functioning veterinary services, while others struggle to provide basic care due to inconsistent policy implementation (Tefera et al. 2024). In underserved areas, this inconsistent application detracts from the veterinary industry's overall efficacy and results in subpar animal health outcomes.

#### 2.9.5 Cultural and Social Barriers

The difficulties in providing veterinary care in Ethiopia are also strongly influenced by social and cultural factors (Desta 2015). In many rural communities, livestock management and animal health care are guided by traditional beliefs, rituals, and indigenous knowledge systems that have been passed down for generations. For example, some communities rely on herbal remedies, spiritual rituals, or divination practices to prevent or treat livestock diseases, rather than seeking modern veterinary interventions. In other cases, certain illnesses are attributed to supernatural causes, such as curses or displeasure of spirits, leading owners to prefer traditional healers over veterinarians. Moreover, livestock may be considered communal or sacred, restricting external interventions or limiting treatments such as vaccinations or medications. These beliefs can result in delayed reporting of disease, reluctance to adopt vaccination programmes, or resistance to culling sick animals, which undermines disease control efforts (Geiger et al. 2020). In addition, low awareness about the benefits of modern veterinary care often reinforces reliance on customary practices, perpetuating preventable disease outbreaks. Overcoming these barriers requires culturally sensitive approaches, including educating livestock owners, demonstrating the value of veterinary interventions, and integrating effective traditional practices with modern veterinary care to ensure community acceptance and participation.

#### 2.9.6 Public Health Interface and Legal Framework for Zoonotic Diseases

The legal framework for handling zoonotic diseases in Ethiopia is established under Proclamation No. 1054/2017 (Animal Disease Prevention and Control Proclamation) and Proclamation No. 267/2002 (Veterinary Practice Regulation), which mandates a multi‐sectoral One Health approach involving the Ministry of Agriculture (MoA), the Ministry of Health (MoH), the Ethiopian Public Health Institute (EPHI), and regional health and agricultural bureaus. Under this framework, notifiable zoonotic diseases including rabies, anthrax, brucellosis, bovine tuberculosis, HPAI, and Rift Valley fever (RVF) require immediate reporting: livestock owners, veterinarians, and CAHWs must report suspected animal cases to district veterinary offices within 24 h, while human cases must be reported by health professionals to district health offices within the same timeframe. The legal framework establishes a joint surveillance and response mechanism through the Technical Working Group (TWG) on Zoonotic Diseases, co‐chaired by the MoA and the MoH, which coordinates outbreak investigation, laboratory confirmation, and response activities.

For confirmed zoonotic outbreaks, the framework prescribes specific stakeholder actions: the MoA (through the AHI) is responsible for animal vaccination (rabies, anthrax), movement restrictions, culling of infected animals with compensation (for HPAI), and public awareness campaigns for livestock owners. The MoH (through EPHI) is responsible for human post‐exposure prophylaxis (rabies PEP), human case management, contact tracing, and health worker training; regional health and agricultural bureaus implement field response activities including ring vaccination, quarantine enforcement, and risk communication; and the EVA and professional bodies support training and public education. However, despite the existence of this legal framework, enforcement is weak due to poor coordination between sectors, limited funding for joint response activities, inadequate laboratory capacity for confirmatory zoonotic testing at regional levels, and low community awareness leading to underreporting. For example, rabies which kills an estimated 2700–3000 Ethiopians annually remains endemic due to incomplete dog vaccination coverage (estimated at <20% in rural areas) and limited access to PEP, despite clear legal mandates for control. Similarly, anthrax and brucellosis continue to circulate unchecked due to inconsistent reporting and lack of integrated human–animal surveillance systems.

### 2.10 Market Access and Economic Factors

Veterinary service delivery in Ethiopia is significantly constrained by challenges related to market access and economic conditions, which directly affect livestock health, productivity, and the broader livestock economy (Tegegne et al. 2010). Geographic barriers, including remote and hard‐to‐reach rural areas, combined with poor transportation infrastructure, limit the ability of veterinarians to reach livestock owners and deliver essential services such as vaccinations, treatments, and diagnostic support (Armson et al. 2020). Even where veterinarians are present, inadequate facilities, insufficient veterinary products, and limited laboratory or pharmacy access reduce the effectiveness of service delivery (Wondie et al. 2024).

Economic and financial constraints further hinder veterinary care. Many livestock owners, particularly in rural areas, cannot afford veterinary services or lack awareness of the benefits of preventive care, which reduces demand and discourages investment in veterinary services. Although government policies and subsidies exist to improve access, their implementation is often inconsistent, and financial support may not reach those who need it most (Desta 2015; Tegegne et al. 2010). Underinvestment in the veterinary sector, both from public and private actors, results in poor infrastructure, limited training opportunities for professionals, and inadequate research and development, making it difficult to attract and retain qualified personnel (Rege and Sones 2022). Overall, market access and economic limitations create a cycle of inadequate service provision: low demand and high costs reduce investment, which in turn diminishes service availability, quality, and reach, particularly in remote areas. Addressing these challenges requires coordinated strategies that improve infrastructure, ensure equitable distribution of veterinary products and services, and strengthen financial support mechanisms to make veterinary care both accessible and economically viable.

### 2.11 Coordination and Collaboration

More cooperation between universities, research organizations, and veterinary clinics is required to promote innovation and information exchange. Opportunities for improvements in veterinary diagnostics and illness management are diminished by the frequent lack of collaborations in the current era (Ahmed et al. 2024). New medications, vaccinations, and diagnostic instruments that are more tailored to Ethiopia's unique needs can be developed through collaboration. Developing more potent vaccines against diseases that are common in the area or producing inexpensive, user‐friendly diagnostic instruments for low‐resource environments are two examples of local research priorities.

The future generation of veterinarians and veterinary paraprofessionals are mostly trained in academic institutions (Cobbold et al. 2020). These universities may guarantee that their curriculum are in line with the practical requirements of the industry by working with veterinary services. This will result in graduates who are well equipped to handle the difficulties they will encounter in the field. For veterinary professionals, ongoing collaboration can help with professional development and continuing education. This is especially crucial in a field that is evolving quickly, where new illnesses, medical advancements, and treatment methods are appearing all the time.

#### 2.11.1 Fragmented Institutional Framework

The shared duty structure operates across four levels: federal (policy formulation, regulation, vaccine production, and reference diagnostics), regional (service oversight, laboratory services, and clinic licensing), district (day‐to‐day service delivery, vaccination campaigns, and disease reporting), and community (CAHWs providing basic first aid and vaccination under supervision). NGOs complement government services in pastoral and remote areas where public infrastructure is minimal, while commercial enterprises focus on clinical services and drug distribution in urban and peri‐urban centres. Coordination occurs through the National Veterinary Technical Committee and the Zoonotic Disease Technical Working Group, though fragmentation and weak information sharing remain significant challenges (Ahmed et al. 2024; Gizaw et al. 2021).

In Ethiopia, NGOs have been instrumental in expanding the availability of veterinary services, particularly in underserved pastoral and remote agropastoral areas (Jilo et al. 2016). CAHWs provide vital preventative and control services (Gizaw et al. 2021). They are frequently trained and supported by NGOs (Ahmed et al. 2024). In regions with inadequate or nonexistent public veterinary infrastructure, more livestock keepers have access to services thanks to these NGO‐sponsored programmes. Additionally, NGOs can serve as catalysts for market‐based models and PPPs that make use of local community resources and expertise. For instance, NGOs play a significant role in animal health interventions alongside governmental organizations and the commercial sector, providing organizational and technical assistance for community‐based veterinary service delivery, according to a stakeholder study by Tesfa et al. (2025).

Despite these efforts, there are still issues with the long‐term viability of NGO‐led veterinary interventions in Ethiopia. Due to a lack of long‐term financial sources, many CAHWs projects fail or drastically reduce when external support from NGOs stops (Fedlu et al. 2019). Inadequate supervision, lack of explicit regulations, and a lack of ongoing professional development to guarantee quality and responsibility are all examples of institutional support for CAHWs (Fedlu et al. 2019). Furthermore, PPP models demonstrate that although NGOs can effectively pilot last‐mile veterinary service delivery approaches, the associated service delivery costs remain high, and donor‐driven interventions are rarely integrated into national veterinary systems (Berhanu et al. 2025). These challenges ultimately undermine the long‐term benefits of NGO contributions toward building a robust, sustainable, and equitable veterinary service infrastructure in Ethiopia.

#### 2.11.2 Weak Communication

Inadequate infrastructure and a lack of coordination among stakeholders are two factors contributing to poor communication in Ethiopia's veterinary industry (Gebremedhin 2015). When we refer to ‘inadequate communication infrastructure’ in Ethiopia's veterinary system, we mean the physical, technological, and organizational systems necessary for timely and effective information exchange among stakeholders. Responses to disease outbreaks and other emergencies are sometimes delayed by a lack of cooperation between federal, regional, and municipal veterinary agencies. Timely exchange of information is hampered in rural and isolated places by inadequate phone networks, poor internet access, erratic cell coverage, and a lack of defined reporting procedures. Furthermore, veterinarians, livestock officers, government organizations, and private stakeholders are unable to efficiently exchange surveillance data, immunization records, and diagnostic results due to the lack of established communication protocols and collaboration platforms (Gizaw and Berhanu 2020). The overall effectiveness of veterinary service delivery is compromised by this combination of inadequate infrastructure and a lack of cooperation, underscoring the necessity of both enhanced stakeholder participation and better communication technologies.

#### 2.11.3 Limited Collaboration Between Public and Private Sectors

Limited collaboration exists between public and commercial entities in veterinary service delivery, despite the growing involvement of the private sector, especially in the areas of drug distribution and clinical services. According to Gizaw et al. (2021), the absence of a well‐defined regulatory structure and incentives for PPPs limits the possibility of fostering synergy between these industries. This lack of cooperation may result in a situation where private services remain neglected in rural and distant areas while being focused in more lucrative urban areas.

NGOs are essential to Ethiopia's veterinary care system, especially in underprivileged areas. However, frequently, their efforts are not in accord with official government goals (Bedane 2016). This lack of coordination may result in inefficiencies, redundant services, and lost chances to combine resources and knowledge. NGOs' actions could also not always coincide with state veterinary policies, which make it more difficult to establish a cohesive and efficient system for the provision of veterinary services.

#### 2.11.4 Lack of ME Mechanisms

Coordination and collaboration in veterinary services are further complicated by the lack of strong ME procedures. It is challenging to evaluate the success of coordinating efforts, spot gaps, and make required improvements without adequate M&E systems. This oversight gap keeps inefficiencies alive and prevents veterinary care delivery from being continually improved (Girma 2016). For example, in pastoralist groups, attempts to coordinate disease prevention and other veterinary interventions may be impeded by customs and mistrust of institutional veterinary services. Culturally sensitive methods and the participation of community leaders in planning and decision‐making procedures are necessary for involving these groups (Tschopp and Kidanu 2024).

## 3 Conclusion and Recommendation

Ethiopia's veterinary services are essential to preserving food safety, safeguarding the health of animals, and assisting the millions of people who rely on livestock and livestock products as a source of income. The effectiveness of the veterinary industry is hampered by several major issues, including poor public awareness, insufficient infrastructure, a lack of veterinary personnel, a slow adoption of state‐of‐the‐art diagnostic tools, and financial limitations. Inadequate disease surveillance and control techniques, uneven policy implementation, and lax enforcement of rules exacerbate these problems. These issues affect not only the country's livestock production and health but also the people's overall economic well‐being, especially in rural and pastoralist areas. To address the challenges hindering effective veterinary service delivery in Ethiopia, several key actions are recommended. There is an urgent need to enhance veterinary infrastructure, particularly in rural and remote areas, while the government should invest in modern diagnostic technologies to improve disease surveillance and response, including implementing standardized operating procedures and ensuring regular audits for quality control. Strategies to increase the number of trained veterinary professionals, especially in underserved areas, should be prioritized, alongside encouraging collaboration between governmental and non‐governmental organizations in research and community service initiatives to ensure that veterinary services are demand‐driven and address the real needs of livestock owners. Initiatives to educate livestock owners on the importance of regular veterinary care and preventative measures should be expanded, with a focus on reaching remote and marginalized communities. Finally, updating and enforcing veterinary regulations, establishing a centralized regulatory authority, and ensuring consistent implementation of policies across all regions are crucial for improving the quality and uniformity of veterinary services.

## Author Contributions

**Abdu Muhammed**: conceptualization, methodology, investigation, writing – original draft, writing – review and editing. **Mitiku Wamile**: writing – review and editing.

## Funding

The authors have nothing to report.

## Ethics Statement

This literature‐based review adhered to high ethical standards, involving no human or animal subjects, with proper citation of sources, unbiased analysis, and no conflicts of interest or funding biases.

## Conflicts of Interest

The authors declare no conflicts of interest.

## Data Availability

The date support the findings of this study are available from the corresponding author upon reasonable request.
